# A chemical screen for medulloblastoma identifies quercetin as a putative radiosensitizer

**DOI:** 10.18632/oncotarget.7980

**Published:** 2016-03-08

**Authors:** Tonny Lagerweij, Lotte Hiddingh, Dennis Biesmans, Matheus H.W. Crommentuijn, Jacqueline Cloos, Xiao-Nan Li, Mari Kogiso, Bakhos A. Tannous, W. Peter Vandertop, David P. Noske, Gertjan J.L. Kaspers, Tom Würdinger, Esther Hulleman

**Affiliations:** ^1^ Department of Pediatric Oncology/Hematology, VU University Medical Center, Amsterdam, The Netherlands; ^2^ Department of Neurosurgery, VU University Medical Center, Amsterdam, The Netherlands; ^3^ Department of Neuro-oncology Research Group, Cancer Center Amsterdam, VU University Medical Center, Amsterdam, The Netherlands; ^4^ Department of Pediatrics, Texas Children's Cancer Center, Baylor College of Medicine, Houston, TX, USA; ^5^ Department of Neurology, Molecular Neurogenetics Unit, Massachusetts General Hospital and Harvard Medical School, Boston, MA, USA

**Keywords:** medulloblastoma, quercetin, radiosensitizer, small molecule, screen

## Abstract

Treatment of medulloblastoma in children fails in approximately 30% of patients, and is often accompanied by severe late sequelae. Therefore, more effective drugs are needed that spare normal tissue and diminish long-term side effects. Since radiotherapy plays a pivotal role in the treatment of medulloblastoma, we set out to identify novel drugs that could potentiate the effect of ionizing radiation.

Thereto, a small molecule library, consisting of 960 chemical compounds, was screened for its ability to sensitize towards irradiation. This small molecule screen identified the flavonoid quercetin as a novel radiosensitizer for the medulloblastoma cell lines DAOY, D283-med, and, to a lesser extent, D458-med at low micromolar concentrations and irradiation doses used in fractionated radiation schemes. Quercetin did not affect the proliferation of neural precursor cells or normal human fibroblasts. Importantly, *in vivo* experiments confirmed the radiosensitizing properties of quercetin. Administration of this flavonoid at the time of irradiation significantly prolonged survival in orthotopically xenografted mice. Together, these findings indicate that quercetin is a potent radiosensitizer for medulloblastoma cells that may be a promising lead for the treatment of medulloblastoma in patients.

## INTRODUCTION

Medulloblastoma, the most common malignant brain tumor in children, accounts for approximately 20% of all intracranial childhood tumors [1]. Currently, patients are stratified into different risk groups based on histological features, clinical criteria, biological profile, and age. In general, treatment consists of surgery and craniospinal radiotherapy, followed by an additional irradiation boost aimed at the primary tumor site (clinicaltrials.gov NCT02066220). Children <3-5 years (age depending on the country) are treated with chemotherapy only [2, 3]. Although such treatment has resulted in remarkable improvement in outcome, this therapy still fails in approximately 30% of medulloblastoma patients [4]. Moreover, such therapy causes severe long-term side effects that significantly impact quality of life, especially in younger children [5–11]. Thus, there is a need for alternative therapies that allow to lower the total dose of irradiation or increase the radiation efficacy. Besides new radiotherapy techniques that limit the radiation doses to surrounding healthy tissues [12–14], new chemotherapeutics that specifically sensitize the tumor to irradiation may provide alternative therapies [15, 16].

Here, we screened a commercially available small molecule library consisting of 960 compounds (ActiTarg-K960) to identify radiosensitizers for human medulloblastoma cells. and identified quercetin (3,3′,4′5,7-pentahydroxyflavone), a flavonoid found in fruits, vegetables and grains, as a potent radiosensitizer. Quercetin treatment at low micromolar concentrations did not affect cell proliferation when used as monotherapy, while the combination with irradiation significantly decreased medulloblastoma cell growth. Importantly, this sensitizing effect was not found on neural precursor cells, or normal human fibroblasts. In addition, quercetin treatment enhanced the *in vitro* sensitivity to radiation of medulloblastoma cell lines in clonogenic survival assays. However, the radiosensitizing effect was not observed in two primary medulloblastoma cell cultures. Finally, we observed that quercetin administration to orthotopically xenograft mice around the time of irradiation significantly prolonged survival. A flow chart, illustrating the experimental design, is available as Supplementary Figure S1. Since quercetin sensitizes medulloblastoma cells in our experiments at radiation doses used in fractionated radiation schemes, and the quercetin concentrations used can easily be achieved by oral administration, we suggest that the use of quercetin should be further evaluated in clinical trials in medulloblastoma patients in the near future.

## RESULTS

### Identification of quercetin as a radiosensitizer for medulloblastoma

**Figure 1:**
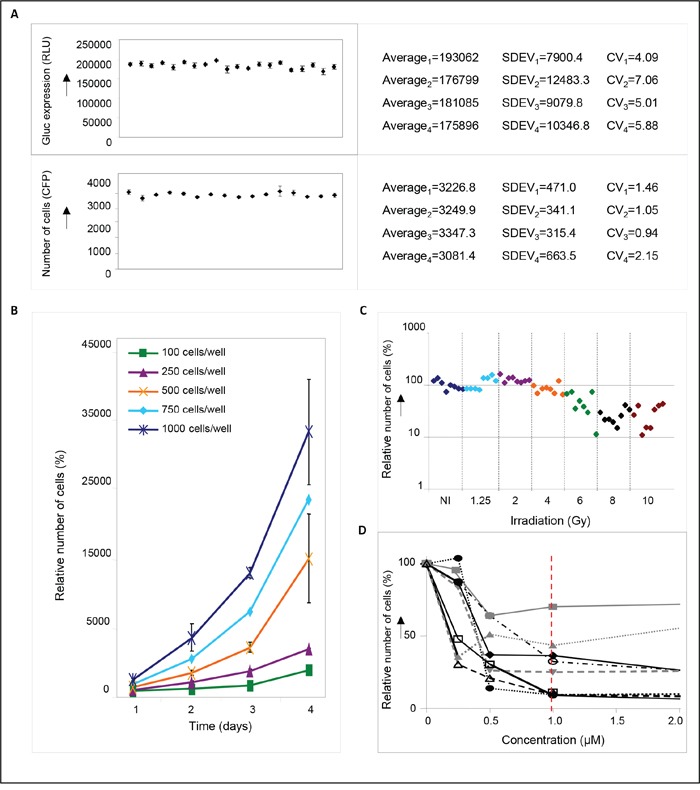
Determination of screening conditions **A.** Reproducibility of Gluc measurements (upper panel) or cell counts as measured by Acumen technology (lower panel). Aliquots of Gluc-containing medium or equal numbers of cells were plated in 96-well plates in quadruplicate, and measured to assess plate-to-plate and well-to-well variation (RLU=relative luciferase units). Data are presented as means ± SD. The corresponding coefficients of variation (CV) are depicted in the right-hand panels. **B.** Growth curves of DAOY medulloblastoma cells. One hundred, 250, 500, 750, or 1000 cells were plated per well and set at 100%. Relative cell numbers were measured at different time points after plating as indicated in the figure. A plating density of 750 cells/well resulted in exponentially growing cells after 4 days of incubation that could be monitored without much variation. Data are presented as means ± SD (n=3). **C.** Graphic representation of the irradiation response in DAOY cells. Growth of non-irradiated (NI) cells is set at 100%. For each irradiation dose 8 samples were measured. **D.** concentration curves of DAOY medulloblastoma cells treated for four days with 0, 0.5, 1, or 2 μM of different drugs. Compounds were chosen randomly from the TimTec library: ST027883 (-■-), ST004727 (-•-), ST053862 (-▲-), ST012157 (-▼-), ST012256 (-♦-), ST029265 (-○-), ST036501 (-□-), ST052055 (-Δ-). A drug concentration of 1 μM was (red dotted line) was used for the final screen.

**Figure 2:**
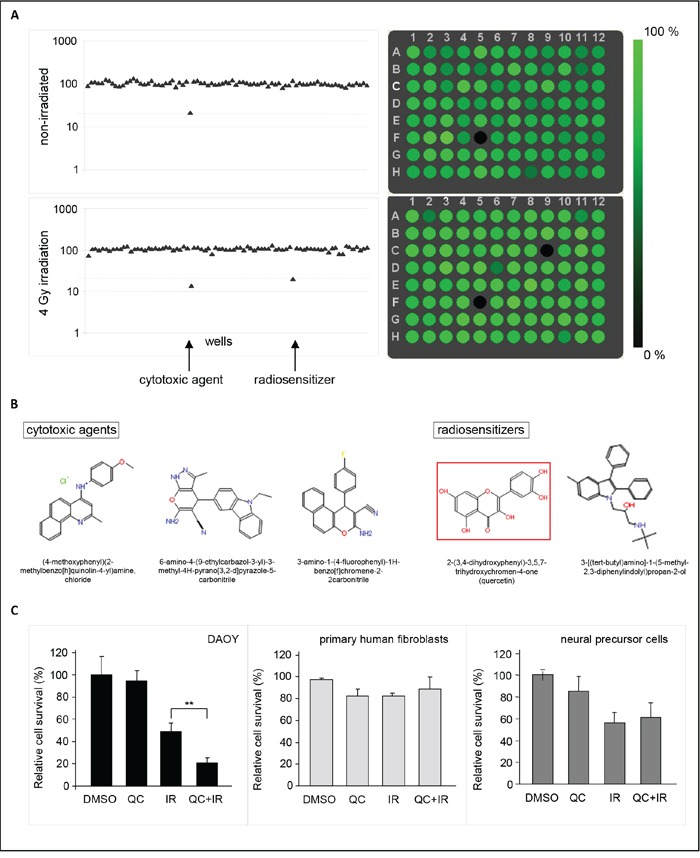
A small molecule screen identifies quercetin as a radiosensitizer in medulloblastoma cells **A.** Example of a scatter plot of Gluc values (left panels) and Acumen read-out (right panels), representing cell survival after treatment of DAOY cells with 1 μM of the ActiTarg-K960 small molecule library in the presence, or absence of irradiation (4 Gy). The fluorescence in the single wells as measured by the Acumen is represented as relative intensities of the color green, where black corresponds with little cells and green with many cells. When using Gluc as a read-out, cell viability was measured by luciferase activity and corrected for the toxicity of the solvent, 0.1% DMSO (set to 100%). A reduction of >75% of cell growth was considered to be significant, as indicated by a dashed line. Each dot represents a single well; in position F05 a cytotoxic agent is identified, in position C09 a radiosensitizer. A representative 96-well plate is shown. **B.** Structure formulae of compounds that induce cell death in DAOY medulloblastoma cells but show limited cytotoxicity on primary human fibroblasts and on C17.2 neuronal precursor cells (NPCs). Two radiosensitizing agents and three compounds that induce cell death independently of irradiation have been identified. **C.** Graphic representation of relative cell survival in DAOY medulloblastoma cells (left panel), primary human fibroblasts (PHF, middle panel), or neural precursor cells (NPC, right panel) after quercetin (QC) treatment and/or irradiation (IR), as extracted from the small molecule screen. Data are presented as means ± SD (n=3). ** p<0.005, Mann-Whitney U test.

In order to enable the identification of novel radiosensitizers for medulloblastoma, a small molecule screen was performed using DAOY medulloblastoma cells that were transduced with a lentiviral *Gaussia* luciferase (Gluc) vector co-expressing the fluorescent ‘Cerulean’ (CFP) reporter [17]. Expression of these genes allowed to monitor cell survival by bioluminescent and fluorescent read-out of cell viability. To optimize screening conditions, the well-to-well and plate-to-plate variation, number of DAOY cells, and the dose of irradiation were determined. When assayed for Gluc luciferase activity, a variation coefficient (CV) of < 7% was observed in four independent experiments (Figure 1A), indicating only minimal variation in pipetting errors, substrate stability and measurement errors. An even better CV of < 2% was observed (Figure 1A) when measured by Acumen technology, where equal numbers of cells were plated and detected by CFP expression. Since both assays allowed to monitor cell viability at different time points after treatment, we optimized our screening conditions – number of cells, dose of irradiation, and drug concentrations – by measuring Gluc secretion or cell numbers in time (Figure 1B-1D). This resulted in a four-day assay, using 750 DAOY cells per well with 4 Gy irradiation. In addition, a drug concentration of 1 μM was chosen, since this showed good results in a pilot experiment using eight different, randomly chosen small molecules (Figure 1D), and yielded positive hits in a drug screen performed previously by our group [18]. To identify putative radiosensitizers, cells were treated with compounds from the ActiTarg-K960 drug library consisting of 960 putative kinase inhibitors, or with 0.1% DMSO as an internal control, either as monotherapy, or in combination with irradiation. A reduction of >75% of cell growth after four days of incubation as compared to the DMSO controls was considered to be significant (Figure 2A). In four separate screens, a total of 23 compounds was identified that consistently inhibited cell growth or sensitized towards irradiation, with 12 compounds inducing cell death independently of irradiation, and 11 compounds functioning as radiosensitizers (Table 1 and Supplementary Figure S2). Cytotoxicity of these 23 compounds was subsequently determined on primary human fibroblasts and on C17.2 neuronal precursor cells (NPCs), to assess the therapeutic window (Table 1). This smaller screen narrowed our list of putative novel compounds for use in medulloblastoma down to five: two radiosensitizing agents and three compounds that have been identified as inducers of cell death in DAOY cells independently of irradiation (Figure 2B). The flavonoid quercetin was among these radiosensitizing compounds. Treatment with quercetin 30 minutes prior to irradiation resulted in a 5-fold reduction in cell growth (~20% cell survival), while treatment with quercetin alone did not significantly affect cell viability compared to cells treated with the solvent DMSO (Figure 2C). Irradiation without addition of quercetin resulted in a 2-fold reduction in cell numbers. As mentioned above, these results were not observed in primary human fibroblasts or neuronal precursor cells (Figure 2C).

**Table 1:** Overview of compounds that induce cell death in DAOY medulloblastoma cells, as identified by a small molecule screen

IUPAC name	structure formula	Molecular weight (Da)	common name	Fibroblasts	Neural Precursor Cells
(4-methoxyphenyl)(2-methylbenzo[h]quinolin-4-yl)amine, chloride	C21H19ClN2O	350,85		OK	OK
6-amino-4-(9-ethylcarbazol-3-yl)-3-methyl-4H-pyrano[3,2-d]pyrazole-5-carbonitrile	C22H19N5O	369,43		OK	OK
3-amino-1-(4-fluorophenyl)-1H-benzo[f]chromene-2-carbonitrile	C20H13FN2O	316,33		OK	OK
5-bromo-4-(4-cyclohexyl-5-phenyl(1,2,4-triazol-3-ylthio))-2-phenyl-2-hydropyri dazin-3-one	C24H22BrN5OS	508,44		OK	+/−
4,5-dichloro-2-[(4-fluorophenyl)methyl]-2-hydropyridazin-3-one	C11H7Cl2FN2O	273,09		+/−	+/−
3-[((1E)-2-(2-pyridyl)-1-azavinyl)amino]-6-methyl-4H-1,2,4-triazin-5-one	C10H10N6O	230,23		+/−	cell death
3-[((1E)-2-(2-pyridyl)-1-azavinyl)amino]-4H-1,2,4-triazin-5-one	C9H8N6O	216,2		+/−	cell death
1-cyclohexylazoline-2,5-dione	C10H13NO2	179,22	N-cyclohexylmalemide	cell death	cell death
4,7-dimethylpyridino[3,2-h]quinoline, oxamethane	C14H14N2O	226,28	neocuproine	cell death	cell death
2,9-dimethylpyridino[3,2-h]quinoline	C14H12N2	208,26	neocuproine	cell death	cell death
5-((2E)-5,5-dichloropenta-2,4-dienoyl)-6-methylpyran-2-one	C11H8Cl2O3	259,09		cell death	cell death
(8-chloro(4H-benzo[e]1,3-thiazolo[5,4-c]thiin-2-yl))naphthylamine	C20H13ClN2S2	380,92		cell death	cell death
2-(3,4-dihydroxyphenyl)-3,5,7-trihydroxychromen-4-one	C15H10O7	302,24	quercetin	OK	OK
3-[(tert-butyl)amino]-1-(5-methyl-2,3-diphenylindolyl)propan-2-ol	C28H32N2O	412,57		OK	OK
5-(4,6-dimethylpyrimidin-2-ylthio)-4-nitrobenzo[c]1,2,5-thiadiazole	C12H9N5O2S2	319,37		+/−	OK
3-(indol-3-ylmethylene)benzo[b]pyran-2,4-dione	C18H11NO3	289,29		+/−	cell death
5-[(1E)-2-(4-bromo-3-chlorophenyl)-2-azavinyl]-2-nitrothiophene	C11H6BrClN2O2S	345,6		+/−	cell death
5-[(1E)-2-(2,4-dichlorophenyl)-2-azavinyl]-2-nitrothiophene	C11H6Cl2N2O2S	301,15		+/−	cell death
5-[(1E)-2-(4-iodophenyl)-2-azavinyl]-2-nitrothiophene	C11H7IN2O2S	358,16		cell death	cell death
6-(tert-butyl)-2-[3-(tert-butyl)-5-bromo-2-hydroxyphenylthio]-4-bromophenol	C20H24Br2O2S	488,28		cell death	cell death
[(5-nitro-2-thienyl)methylene]methane-1,1-dicarbonitrile	C8H3N3O2S	205,2		cell death	cell death
9-((1E)-2-nitrovinyl)anthracene	C16H11NO2	249,27		cell death	cell death
di2,3,4,5,6-pentafluorophenyl ketone	C13F10O	362,13		cell death	cell death

Chemical compounds that repetitively induced cell death (upper panel) or functioned as radiosensitizers (lower panel) are represented. NPC=neural precursor cells; OK= no cell death as compared to control, +/− = ≤ 50% cell death.

### Quercetin sensitizes medulloblastoma cells to radiation *in vitro*

**Figure 3:**
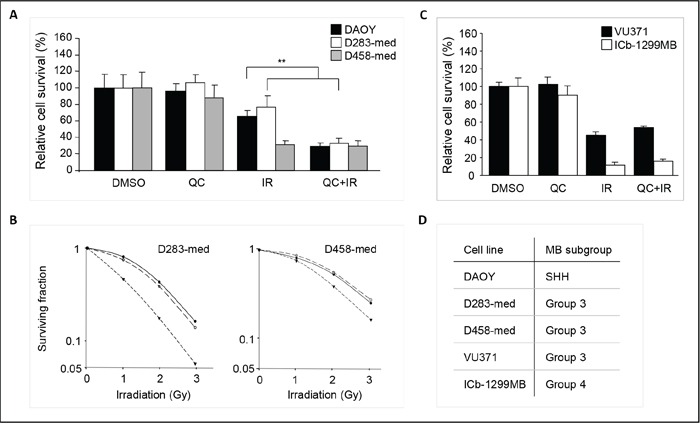
Quercetin sensitizes towards irradiation in a panel of medulloblastoma cells **A.** Graphic representation of relative cell survival of DAOY, D283-med, and D458-med cells after 4 days of quercetin treatment in the presence or absence of irradiation (4 Gy). Cell numbers were determined by visual counts, using a Bürker hemacytometer. Vehicle treated cells are set at 100%. Data are presented as means ± SD (n=3). ** p<0.005, Mann-Whitney U test. **B.** Clonogenic survival of D283-med (left panel) and D458-med (right panel) medulloblastoma cells, 14 days after irradiation (0-3Gy). Cells were treated with 0 μM (-•-), 0,5 μM (-○-), or 1 μM (-▼-) quercetin 30 minutes prior to irradiation. A representative experiment is shown. **C.** Graphic representation of relative cell survival of VU371 and ICb-1299MB cells after 4 days of quercetin treatment in the presence or absence of irradiation (0.7 Gy). Cell viability was determined by Cell Titer Glo assay. Vehicle treated cells are set at 100%. Data are presented as means ± SD. **D.** Molecular subgroup classification of DAOY, D283-med, D458-med, VU371, and ICb-1299MB cells. Expression levels of subgroup classifiers WIF1, SFRP1, NPR3, and KCNA were determined by qRT-PCR to determine to which subgroups the used medulloblastoma cells belong.

Since quercetin has been reported to effectively cross the blood-brain-barrier [19], we hypothesized that this compound could be an attractive agent for the treatment of medulloblastoma. Therefore, we investigated if similar effects could be observed in additional medulloblastoma cell lines. D283-med, D458-med, and DAOY cells were incubated with 1 μM quercetin 30 minutes prior to irradiation, and cell numbers were determined at 4 days after treatment (Figure 3A). A radiosensitizing effect of quercetin was observed in DAOY and D283-med cell lines, while quercetin treatment by itself did not inhibit cell proliferation in any of the tested cell lines. To further confirm the radiosensitizing potential of quercetin, dose-dependent clonogenic survival assays were performed. Although DAOY cells are reported to be clonogenic [20, 21], in our hands DAOY cells appeared unfit for these clonogenic experiments, since they did not form clones. However, treatment of D283-med and D458-med medulloblastoma cells with 0.5 μM or 1 μM quercetin showed radiosensitization in both cell lines (Figure 3B), even at radiobiologically relevant irradiation doses of 1 Gy or 2 Gy used in fractionated radiation schemes. These results strengthen our hypothesis that quercetin is a putative radiosensitizer for medulloblastoma. Next, we tested the radiosensitizing effect of quercetin on two primary medulloblastoma cell cultures, VU371 and ICb-1299MB [22]. Similar as for the cell lines, cells were treated with 1 μM quercetin 30 minutes prior to irradiation, and viability was determined four days after treatment (Figure 3C). Again, quercetin by itself did not reduce cell viability, however, these cells are highly sensitive to radiation (data not shown). Therefore, cells were irradiated with the lowest technical dose possible, 0.7 Gy, which already significantly impaired viability. We did not observe an enhanced radiation response in combination with quercetin in these cells. Since medulloblastomas have been subclassified into four molecular subgroups [23], we determined to which subgroups the used cell lines belong. Expression of four subgroup classifier genes was assessed by qRT-PCR, which was previously described by Zhao *et al*. [22] (Supplementary Figure S3A+B). DAOY was identified as a SHH-group like medulloblastoma while D283-med, D458-med, and VU371 belonged to the group 3 subtype (Figure 3D and Supplementary Figure S3A+B). ICb-1299MB was previously classified as a group 4 medulloblastoma [22].

### Quercetin treatment improves radiation efficacy in a xenograft tumor model

**Figure 4:**
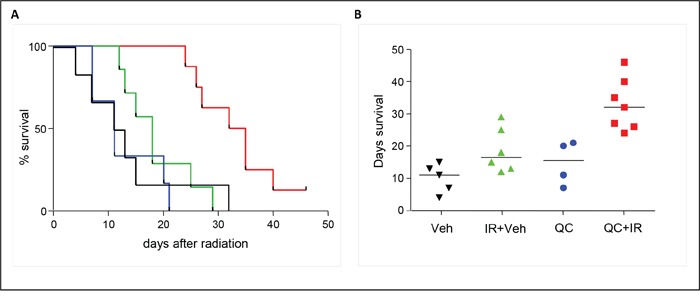
Effect of quercetin treatment in combination with irradiation on survival in a xenograft mouse model **A.** Kaplan-Meier survival analysis of medulloblastoma-bearing mice treated with quercetin (blue line), vehicle (5% DMSO – black line), vehicle and irradiation (green line), and quercetin in combination with irradiation (red line). A significant survival extension of the group that received ionizing radiation in combination with quercetin as compared to the vehicle-treated group (p=0.0052), the quercetin group (p<0.0001), or the group that only received radiotherapy (p=0.002) was observed. **B.** Graphical representation of survival in days; medians are indicated as horizontal lines.

To further evaluate if quercetin can function as a novel agent in the treatment of medulloblastoma, we investigated the effect of this compound in an *in vivo* setting, using an orthotopic xenograft mouse model. Therefore, luciferase-expressing D283-med cells were implanted stereotactically into the left cerebellar hemisphere of nude mice. In two independent experiments, 31 out of 36 mice developed primary tumors, as judged by bioluminescent imaging (BLI) three weeks after implantation. These mice were then randomly assigned into four groups, and treated with vehicle (5% DMSO) or quercetin, either alone or in combination with irradiation. Since quercetin has a half-life of only 20 minutes *in vivo* [24], six consecutive injections with quercetin (100 mg/kg) were given at time points around the time of irradiation (60, 30 and 5 minutes before irradiation plus 30 and 60 minutes after irradiation and 24 hours after irradiation). Quercetin treatment did not induce any neurological symptoms or other adverse events. Tumor growth was monitored twice a week by BLI. In addition, the mice were monitored daily for discomfort and weight loss. Although the BLI signal of the tumors did not differ between the various groups, survival analysis indicated a significant extension of the group that received ionizing radiation in combination with quercetin as compared to the vehicle-treated group (p=0.0052), the quercetin group (p<0.0001), or the group that only received radiotherapy (p=0.002; Figure 4A). All animals treated with the combination of quercetin and irradiation survived for more than 24 days after treatment, with a median survival time of 32 days. Animals that did not receive irradiation (either vehicle- or quercetin-treated) had a median survival of only 12-17 days (Figure 4B).

## DISCUSSION

Medulloblastoma therapy still fails in approximately 30% of patients and is often accompanied by severe long-term sequelae. Thus, there still is a need for alternative therapies that allow to lower the total dose of irradiation, reducing the long-term side effects, and/or increase the radiation efficacy. We show here that the flavonoid quercetin can sensitize medulloblastoma cells to irradiation, and that administration of quercetin during radiotherapy significantly improves survival in mice harboring medulloblastoma.

Quercetin was identified as a ‘ready-to-use’ radiosensitizer in a small molecule screen for medulloblastoma cells, using two independent read-out systems. In this screen 23 compounds were repeatedly identified that could inhibit cell growth (n=12), or sensitize towards irradiation (n=11). Strikingly, two of the compounds that induced cell death independently of irradiation had similar structures, as did three of the 11 compounds that functioned as a radiosensitizer (nitrophenes, Supplementary Figure S2). However, most of those drugs also induced cell death in primary human fibroblasts or neuronal progenitor cells, rendering them unfit for the development of novel and tumor-specific therapies. Unlike those compounds, quercetin did not affect the proliferation of neuronal precursor cells or normal human fibroblasts, nor showed any toxicity in the absence of ionizing radiation. This is in concordance with previous studies that report selective activity of quercetin as a sensitizer to chemotherapeutics on cancer cells, but not in normal cells, even though higher quercetin concentrations (ranging from 5-200 μM) were used in these studies [25–27]. Moreover, quercetin has been shown to enhance radiation-induced cell death in rat hepatoma cells [28, 29]. Importantly, the low micromolar concentrations used in our experiments are in the range of the plasma concentrations that can be reached in humans and are considered to be safe [30, 31]. Quercetin is found in a broad range of fruits and vegetables such as apples, onions and tomatoes, and present in plasma at the nanomolar range (<100 nM) through our dietary intake, but micromolar concentrations have been reported after supplementation [32, 33].

The observation that the concentrations needed for therapeutic benefit can be easily achieved by oral administration, and the fact that quercetin is cheap and readily available, renders this flavonoid an interesting option for the treatment of children with medulloblastoma. Another motivation to consider quercetin as a radiosensitizer for this type of brain tumors, is the previously reported observation that quercetin can pass the blood-brain barrier (BBB) [34, 35]. The BBB is a natural boundary between circulating blood and cerebrospinal fluid that protects the brain from toxins and potentially harmful substances (reviewed by Agarwal *et al*. [36]). Although beneficial under normal circumstances, the presence of this BBB constitutes a major obstacle for drug delivery in the treatment of brain tumors. Importantly, quercetin has not only been shown to pass the BBB in *in vitro* systems, but has also been shown to accumulate in the brain after oral administration in rats [19]. Moreover, quercetin has been shown to function as a neuroprotective agent, both *in vitro* and *in vivo* following ischemia, trauma, or other forms of induced brain damage [19, 37–39]. Of particular interest in the context of medulloblastoma treatment is the observation that administration of quercetin can improve learning and memory deficits in animals that were subjected to brain damaging agents [40–42].

At low concentrations, quercetin is a potent anti-oxidant that can scavenge free radicals and bind transition metal ions. The neuroprotective effect of quercetin has been suggested to be due to this anti-oxidative property. However, at higher cellular concentrations (40-100 μM), quercetin exerts pro-oxidative effects [52–54]. At doses above 100 μM alternative mechanisms, such as modulation of signal transduction pathways or effects on gene expression have also been reported [55]. Quercetin is extensively metabolized through glucuronation, sulfation and/or methylation. Metabolites were detected in urine, plasma and liver homogenate of mice fed with quercetin [43]. Formation of different metabolites may explain concentration dependent effectiveness, as O-methylated metabolites are less protective against peroxide-induced damage than quercetin [44] whereas glucurono-sulfo metabolites are four times more potent in inhibition of LDL oxidation [45]. The relative contribution of the different metabolites to the radiosensitization of the medulloblastoma cells is currently not known. To test which are the major metabolites which would reach the tumor *in vivo*, bioavailability in the brain could be studied in preclinical experiments by the use of cerebral microdialysis [46] or tissue collection at several endpoints. To measure the effects in humans, a phase 0 study could be designed to test the concentration of quercetin and its metabolites by microdialysis [47], biopsies or surgically removed tumor tissues. Quercetin can modulate the activity of many kinases and other enzymes (reviewed by Russo *et al*. [48]), which may explain the diversity of its actions. Besides neuroprotective properties, quercetin has been described to prevent cardiovascular diseases [49], to function as a chemopreventive agent [50], to have anti-proliferative and growth-suppressing effects [51], to induce senescence and autophagy [52, 53], and to have anti-inflammatory and anti-angiogenic activities [54, 55]. Each property or action appears to depend on the dose and model system used. Of note are the inhibition of the Wnt/β-catenin pathway, which plays an important role in one of the four medulloblastoma subgroups [23], and the inhibition of the Hepatocyte Growth Factor (HGF)-induced cell migration in medulloblastoma by quercetin [56]. Furthermore, quercetin has been reported to promotes cell apoptosis and in human glioma cell lines by the suppression of PI-3-kinase-Akt and ERK signalling pathways [57, 58] and induce radiosensitization in tumor cells other than medulloblastoma by targeting the ATM-mediated pathway, which is critical in the DNA damage response [59]. Which pathway or mechanism is involved in the radiosensitization of medulloblastoma cells by quercetin is not exactly known at the moment.

Four distinct molecular subtypes of medulloblastoma have been identified [23]. Subclassification of the cell lines used in this study showed that the SHH-group like and group 3 subtypes were represented, indicating. that the mechanism underlying the improved radiation response caused by quercetin may be subtype-independent. The primary cell cultures used in this study belong to the group 3 and group 4 subtypes. We did not detect a radiosensitizing effect of quercetin in these cells, since these cells are highly radiosensitive *in vitro* and there is no window in which quercetin can exert its effect. This was also seen in D458-med cells, which show high sensitivity to radiation. Since the established medulloblastoma cell lines used in our experiments may be genetically divergent from *de novo* tumors, it It would be of interest to examine the efficacy of quercetin in radioresistant primary medulloblastoma cell cultures that resemble the original tumor. In addition, the response of primary cell cultures *in vitro* may not necessarily represent the response in the cerebellar environment. Therefore, we are setting up more primary cell lines of the different subtypes and use these to develop orthotopic mouse models to study the effect of quercetin as a radiosensitizer in medulloblastoma, in an environment that is more comparable to that of the original tumor. However, the results presented here indicate that quercetin functions as a potent radiosensitizer in medulloblastoma cells at easily achievable concentrations, providing a promising lead for treatment of children with this type of brain tumor.

## MATERIALS AND METHODS

### Cell culture and lentiviral infections

Mouse C17.2 neural precursor cells [60], human primary fibroblasts (American type culture collection; ATCC) and D283-Med, D458-Med, DAOY medulloblastoma cell lines were cultured at 37°C in a 5% CO_2_ humidified atmosphere in DMEM plus 10% fetal calf serum, 100IU/ml penicillin and 100μg/ml streptomycin (PAA Laboratories GmbH, Austria). Pools of D283-med FM/GC or DAOY FM/GC cells were generated by infection with lentiviral vectors, expressing the reporter gene combinations *Firefly* luciferase/mCherry (FM) or *Gaussia* luciferase/Cerulean (GC), as described previously [17]. Fluorescence microscopy was used to assess the success rate of transductions and cell viability. For intracranial injections, cells were harvested and suspended in PBS at a concentration of 1 × 10^8^ cells/ml. The cells used in this study were not authenticated.

Primary cell culture VU371 was derived from tumor tissue, surgically removed from a patient diagnosed with medulloblastoma at the VU University Medical Center. Informed consent was obtained according to institutionally approved protocols. ICb-1299MB is a patient derived orthotopic xenograft mouse model of group 4 medulloblastoma [61] and the xenograft cells were kindly provided by Dr. Xiao-Nan Li and Dr. Mari Kogiso (Baylor College of Medicine, Houston, TX, USA). VU371 and ICb-1299MB were cultured at 37°C in a 5% CO_2_ humidified atmosphere in NBM (NeuroBasal Medium)(Invitrogen) supplemented with neural stem cell supplement (NSCS), N2, stable glutamine (PAA), epidermal growth factor (EGF), and basic fibroblast growth factor (bFGF)(PeproTech).

### Chemicals

The ActiTarg-K960 chemical library, consisting of 960 putative kinase inhibitory compounds, was obtained from TimTec (Newark, Delaware, USA). Ninety-one percent of these compounds conform to four Lipinski criteria and 97% to three Lipinski criteria, suggesting they have desirable pharmacologic properties [62]. Quercetin dihydrate (Calbiochem, USA) was dissolved in dimethyl sulfoxide (DMSO; Sigma-Aldrich, USA), with a final DMSO concentration of 0.1% for *in vitro* experiments or a final DMSO concentration of 5% for *in vivo* experiments.

### Chemical library screen

Seven hundred and fifty DAOY cells were plated per well in 96-well plates. The next day, cells in each well were treated with a different compound from the ActiTarg-K960 library at a 1 μM concentration. Treatment was performed with drugs in paired 96-well plates, where one plate was exposed to 4 Gy in a Gammacell^®^ 220 Research Irradiator (MDS Nordion, Canada) 30 minutes after addition of the compounds, and the other plate was a non-irradiated control. Four days later, cell survival was evaluated by measuring Gaussia luciferase (Gluc) activity [18], or by means of the Acumen ^e^X3 laser scanning cytometer (TTP LabTech, UK). Results were analyzed using Acumen Explorer software, calculating the percentage survival for each compound tested with the assay. Robustness of the assays was determined by calculating the Z’ factor as described by Zhang *et al*. [63], where Z’=0.84 for the Gluc assay and Z’=0.56 for the Acumen screen.

### Survival assays

Responsiveness of D283-med, D458-med (1,000 cells/well, or 1 × 10^4^ cells/flask), or DAOY (1,500 cells/well) medulloblastoma cells to quercetin (1 μM) was determined in a cell proliferation assay by use of the Acumen ^e^X3 laser scanning cytometer as described above, and confirmed by cell counts using a Bürker hemocytometer. Viability of primary cells, VU371 and ICb-1299MB (2,000 cells/well), after treatment with 1 μM quercetin and 0.7 Gy irradiation was determined with Cell Titer Glo assay (Promega) according to manufacturer's protocol. In addition, a clonogenic assay was performed with different doses of irradiation (0-3 Gy). Therefore, exponentially growing D283-med and D458-med cells were plated in triplicate in 6-well plates at concentrations ranging from 200-2,000 cells/well, and grown for 14 days in MethoCult^®^H4001 with 0, 0.5, or 1 μM quercetin. Colonies were counted visually and plating efficiency (PE) was calculated by dividing the number of colonies counted by the number of cells plated. Surviving fractions (SF) were then calculated by dividing the PE by the PE of the non-irradiated control per drug concentration. Duplicate experiments were performed for each cell line. Analysis of inhibitory concentrations was performed using SigmaPlot 11.0 (Systat Software, Inc. San Jose, CA, USA).

### qRT-PCR

Quantitative RT-PCR (qRT-PCR) analysis was performed to determine expression of the medulloblastoma subgroup classifiers WIF1, SFRP1, NPR3, and KCNA in medulloblastoma cell lines and a normal cerebellum sample. Total RNA was isolated using the TRIzol RNA isolation protocol (Invitrogen, Carlsbad, CA, USA) and equal amounts of RNA were converted to cDNA using the Omniscript kit (Qiagen). Primer sequences for WIF1, SFRP1, NPR3, and KCNA transcripts were previously described by Zhao *et al*. [22] and primers were manufactured by Biolegio (Nijmegen, The Netherlands). Gene expression of the subgroup classifiers in medulloblastoma cells was normalized to GAPDH expression levels and the Ct values were used to calculate the relative fold difference in mRNA levels (ΔΔCt method) compared to normal cerebellum.

### D283-med orthotopic xenograft mouse model

Female athymic nude-Fox1nu mice (age 8-10 weeks; Harlan, Horst, The Netherlands) were maintained in accordance with the guidelines and regulations set out by the VU University committee on research animal care. For intracranial injections, mice were anesthetized with 2.5% isoflurane in oxygen, and a volume of 5 μl (0.5 × 10^6^ D283-med FM-GC cells) was injected stereotactically into the cerebellum at a rate of 2 μl per minute, using a Hamilton 10 μl syringe with a 26G needle. Coordinates for injection were determined according to the mouse brain atlas [64]: 2.0 mm lateral, 2.5 mm ventral of lambda, and at a 2.0 mm depth. Before start of the treatment (three weeks after injection of the tumor cells) tumor engraftment was determined by measuring *Firefly* luciferase (Fluc) activity. Based on this activity, mice were randomized into four treatment protocols: vehicle (5% DMSO), quercetin, DMSO/irradiation (4 Gy), and quercetin/irradiation (4 Gy). Mice without tumor engraftment (Fluc activity <80,000 photons/sec at three weeks after implantation) were excluded from the experiment. Quercetin (100 mg/kg) was administered intraperitoneally at six time points: 30 and 60 minutes before-, or after irradiation, 0 hours, and 24 hours after irradiation. Mice not receiving quercetin were treated with the same volume of DMSO dissolved in PBS, at the same time intervals as the quercetin treated mice. To enable precise positioning of the radiation beam on the head and neck area of the mice, the animals were anesthetized with ketamin/xylazin. Mice that were not irradiated were anesthetized just before the zero hours’ time point. Tumor growth was monitored semi-weekly by bioluminescent imaging (BLI). In short, 150 μl D-luciferin (0.03 g/L, Gold Biotechnoloby, St. Louis, USA) was injected intraperitoneally and 10 minutes after administration mice were anesthetized with isoflurane inhalation anaesthesia, positioned in the IVIS camera and the bioluminescence signal was determined with the IVIS Lumina CCD camera. In addition, the mice were monitored daily for discomfort and weight loss. When moderate to severe symptoms were present (weight loss of >20% or severe neurological deficits), animals were sacrificed and brains were removed and formalin-fixed.

### Statistical analysis

A coefficient of variation (CV) and Z’ factor were calculated to assess the reproducibility and robustness of the small molecule screens, as described by Zhang *et al*. [63], where CV=SD/μ and Z’ = 1-(3σ_c+_+3σ_c-_)/Iμ_c+_-μ_c-_I. Statistical significance of treatment was assessed using the Mann-Whitney U test. Kaplan-Meier survival curves were generated with GraphPad Prism 5. Median survival of the groups was calculated and survival curves were compared with the Log-rank (Mantel-Cox) test. The *p* values <0.05 were considered statistically significant.

## SUPPLEMENTARY FIGURES


