# Supplementary material for: A chemical screen for medulloblastoma identifies quercetin as a putative radiosensitizer
Source: Oncotarget. 2016 Mar 8;7(24):35776–88. doi: 10.18632/oncotarget.7980 (PMC5094961; doi:10.18632/oncotarget.7980)
Supplement: Supplementary file 1 [file oncotarget-07-35776-s001.pdf]

A chemical screen for medulloblastoma identifies quercetin as a putative radiosensitizer

SUPPLEMENTARY FIGURES

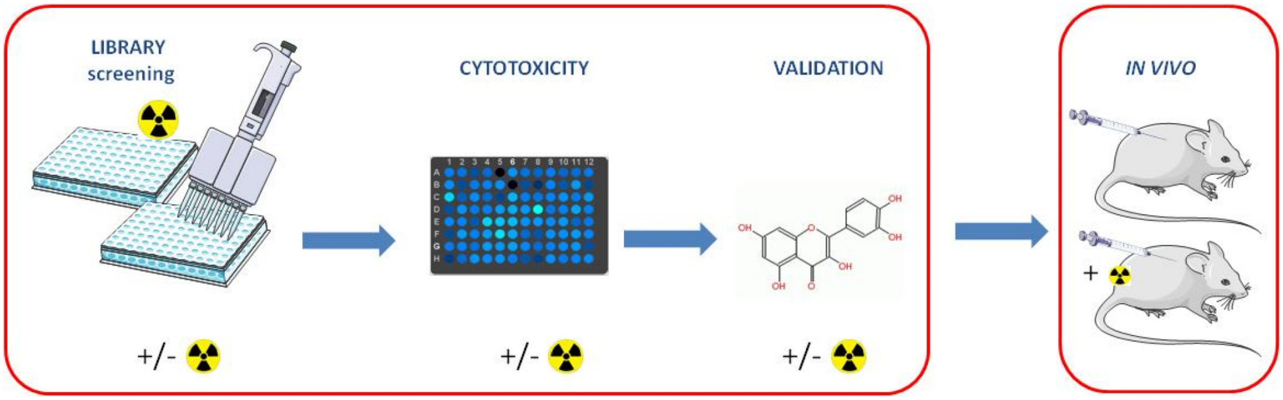

Supplementary Figure S1: Schematic layout of the study design. The ActiTarg-K960 library was screened for radiosensitizers with the DAOY medulloblastoma cell line. Twenty-three compounds were identified and further tested for toxicity on fibroblasts and neuronal precursor cells. The radiosensitizing effect of quercetin was further validated with other medulloblastoma cell lines. Finally, the radiosensitizing potential was tested in a medulloblastoma mouse model.

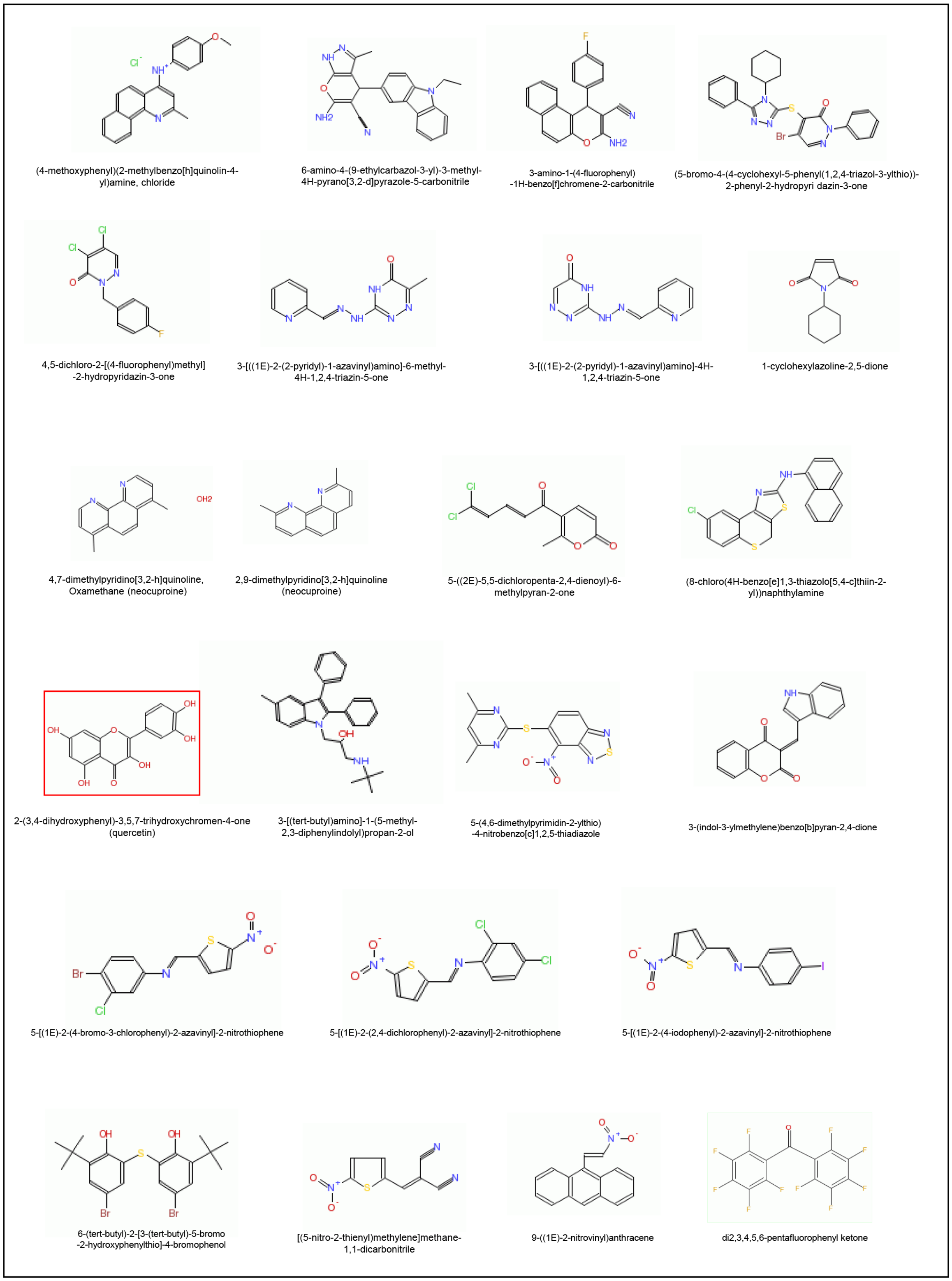

Supplementary Figure S2: Structure formulae of compounds that induce cell death in DAOY medulloblastoma cells, as identified by a small molecule screen. Chemical compounds that repetitively induced cell death (upper twelve) or functioned as radiosensitizers (lower structures) are represented.

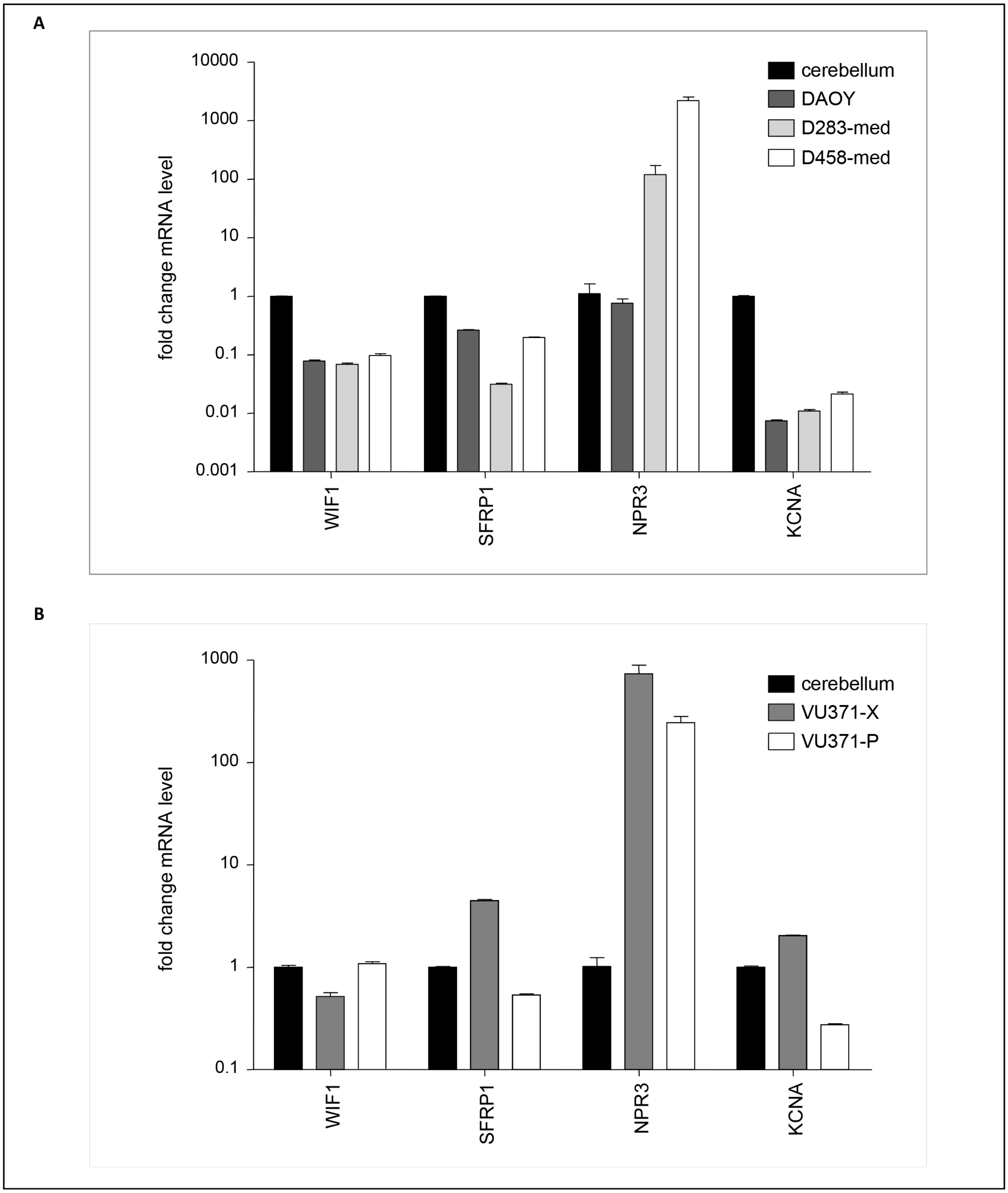

Supplementary Figure S3: Expression levels of subgroup classifiers WIF1, SFRP1, NPR3, and KCNA in medulloblastoma cells compared to normal cerebellum. A. expression levels of WIF1, SFRP1, NPR3, and KCNA in DAOY, D283-med, and D458-med compared to normal cerebellum. DAOY was identified as a SHH medulloblastoma, D283-med and D458-med were identified as group 3 medulloblastomas. **B.** expression levels of WIF1, SFRP1, NPR3, and KCNA in VU371 cells from a xenograft tumor (VU371-X) and VU371 parental cells from the original tumor (VU371-P) as compared to expression in normal cerebellum. VU371 was identified as a group 3 medulloblastoma. The subgroup type was preserved after serial transplantation of tumor cells in the cerebellum of mice.
